# No evidence for loss of short-wavelength sensitive cone photoreceptors in normal ageing of the primate retina

**DOI:** 10.1038/srep46346

**Published:** 2017-04-12

**Authors:** Tobias W. Weinrich, Michael B. Powner, Aisling Lynch, Ravi S. Jonnal, John S. Werner, Glen Jeffery

**Affiliations:** 1 Institute of Ophthalmology; University College London, London, EC1V 9EL, United Kingdom; 2 Division of Optometry and Visual Sciences; City, University of London, London, EC1V 0HB, United Kingdom; 3 Department of Ophthalmology and Vision Science; University of California, Davis, Sacramento, CA 95817, United States of America

## Abstract

In old world primates including humans, cone photoreceptors are classified according to their maximal sensitivity at either short (S, blue), middle (M, green) or long (L, red) wavelengths. Colour discrimination studies show that the S-cone pathway is selectively affected by age and disease, and psychophysical models implicate their loss. Photoreceptors have high metabolic demand and are susceptible to age or disease-related losses in oxygen and nutrient supply. Hence 30% of rods are lost over life. While comparable losses are not seen in cones, S-cones comprise less than 10% of the cone population, so significant loss would be undetected in total counts. Here we examine young and aged primate retinae stained to distinguish S from M/L-cones. We show there is no age-related cone loss in either cone type and that S-cones are as regularly distributed in old as young primates. We propose that S-cone metabolism is less flexible than in their M/L counterparts, making them more susceptible to deficits in normal cellular function. Hypoxia is a feature of the ageing retina as extracellular debris accumulates between photoreceptors and their blood supply which likely impacts S-cone function. However, that these cells remain in the ageing retina suggests the potential for functional restoration.

With ageing and disease there is cell loss and reduced function. Age-related changes are partly linked to metabolic rate with a high metabolic rate associated with a faster pace of ageing^1^. The CNS has a very high metabolic rate^2^, and within this the retina has the greatest stress due to the energy demands of photoreceptors^3^. Consequently, there is significant age related photoreceptor loss. Within the rod population there is around a 30% loss with normal ageing in human and rodent^4^,^5^. In humans and old world primates cone photoreceptors are classified according to their maximal sensitivity at either short (S, blue), middle (M, green) or long (L, red) wavelengths. There are key differences between M/L cones and S-cones. S-cones constitute less than 10% of the total cone population whereas the other two types account each for around 45%. S-cones have distinct anatomical and physiological properties compared to M/L cones^6^,^7^. Also, colour discrimination studies show that the S-cone pathway is selectively affected by age and disease^8^,^9^,^10^,^11^. The ageing effect was thought to relate to brunescence of the lens as this would selectively absorb shorter wavelengths^8^. However, there are also significant changes in S-cone timing with age that are not related to lens brunescence^12^. Psychophysical models implicate differential S-cone loss with age, which would represent the most parsimonious explanation for reductions in their function. However, because they only form such a small percentage of the total population, counts of total cone numbers are unlikely to reveal any significant changes in their S-cone population.

Here we examine old world primate retinae from young and old animals stained to reveal S- and M/L-cone populations to determine patterns of age-related cell loss.

## Results

Retinae were acquired from young and old Mauritian *Macaca fascicularis* from a long established breeding colony. These old world primates have retinae highly similar to those in humans^13^, with a well-developed central region that includes a fovea and trichromatic vision^14^,^15^. Further, photoreceptor function measured psychophysically is also very similar between humans and this species of old world primate^16^,^17^. Animals were grouped into young (6 years) and old (17 years). At 6 years eye size is fully mature. At 17 years animals appear physically old and have clear signs of retinal ageing including extra-cellular deposition of lipofuscin^18^,^19^. Primates in the source colony rarely lived beyond 20 years. Macroscopic and microscopic examination confirmed that all retinae used were normal.

**Figure 1:**
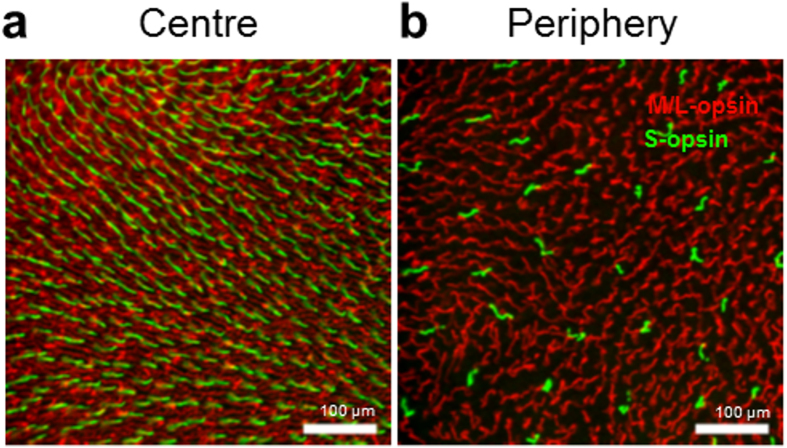
An example of changes in cone density across the temporal retina. The spatial distribution of cones stained for M/L-opsin (red) and S-opsin (green) from a young animal. (**a**) Was taken in the temporal parafovea (1.3 mm eccentricity). (**b**) Was taken in the temporal periphery (10 mm eccentricity). There was a clear gradient in cone density across this axis, but no obvious differences in cone density between young and old animals.

**Figure 2:**
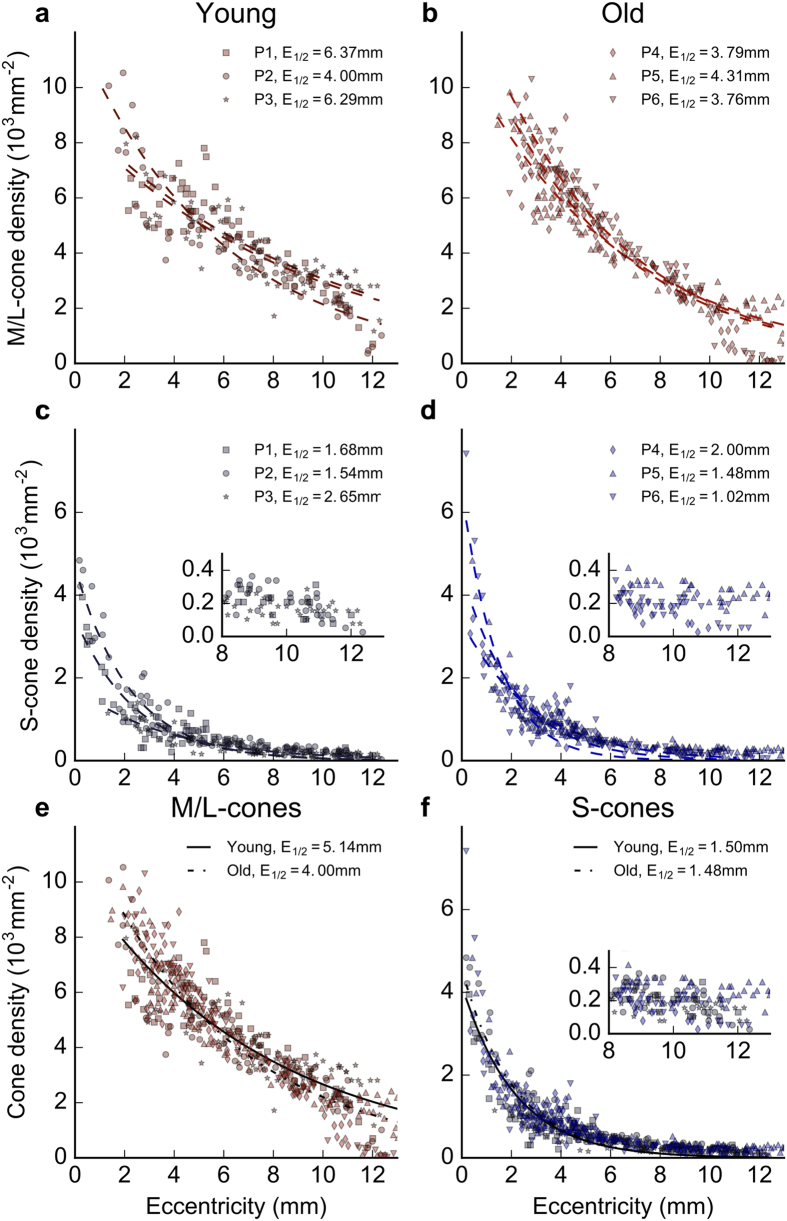
Cone density and eccentricity in young and old animals. Densities of M/L- and S-cones in young and old macaques extending from the central retina to 13 mm temporal periphery. (**a**) The densities of M-/L-cones in 3 young (6 years old) animals. (**b**) Corresponding data for 3 old (17 years old) macaques. Data are not provided for the central most location as cell density was too great for reliable counting. (**c**) Counts of S-cones over exactly the same region in 3 young animals. (**d**) Counts of S-cones over the same region in 3 old primates. In each case patterns are very similar for the two age groups. (**e**) and (**f**) Density of the two age groups are overlaid, for L-/M-cones and S-cones, respectively. (**e**) Densities for M-/L-cones in young and old primates with regression lines for the two populations. Data sets for the two ages match closely except in the far periphery. (**f**) Densities for S-cones in young and old primates with regression lines for the two populations. Again these match closely. Taken together the data in the 6 graphs show that there is no difference between young and old primates in terms of cone densities. Because of the low S- cone density in the periphery, their densities have been represented twice at different scales in the regions beyond 7 mm eccentric. The first is on the same scale as other counts (Fig. 2a and b) and the second is on an expanded Y axis to provide a clearer picture of their distribution.

Flat mounted retinae were uniformly stained with two antibodies, one for S-cone opsin and the other for the combined M/L-cone opsin. The distribution of labelled cones conformed to previous studies with a significant gradient between the central macular and the peripheral retina^15^,^20^ as shown in Figs 1 and 2. Retinae were systematically mapped from the central macula out toward the temporal periphery at approximately 100 locations per retina in a region 0.1 mm^2^ in each of 6 retinae, 3 from each age group.

Figure 2 shows eccentricity-dependent variation in cone density, grouped by age and cone spectral class. Figure 2a and b are M/L-cone densities from the two age groups, and Fig. 2c and d are S-cone densities in each group over the same region. Counts for M/L cones do not extend into the central macula because cell density here was too great for accurate counting. At any given retinal eccentricity, the variance in M/L-cone density appears greater among young animals than old, although this was less than the variability reported for the total cone population in humans^21^. Further, in the young animals the variability was largely due to data from one individual. However, overall aggregate dependence of M/L-cone density on eccentricity is similar in the two age groups (Fig. 2a and b). Plots for S-cone densities in young and old primates over the same regions are again very similar with the steepest decline in density occurring over the 4 mm closest to the fovea (Fig. 2c and d). There is no evidence for a difference in S-cone cell numbers between the two age groups.

Figure 2e and f show cone densities for both young and old animals, grouped by cone spectral class. Within each spectral class, data from young and old animals were fitted separately with the function *D(E*) = *D*_*0*_*e*^−*λE*^, using nonlinear least-squares regression. *D(E*) is the cone density at eccentricity *E, D*_*0*_ is the maximum density, and *λ* is the decay constant. Permutations of *λ* such as time constant (*τ* = *1/λ*) and half-life (*E*_*1/2*_ = *(ln 2)/λ*) describes the rate of decay. The half-life represents the eccentricity at which cone density reaches half its maximum. For M/L-cones, there was a small difference in half-life between young and old animals, with *E*_*1/2*_ = *5.1* *mm and E*_*1/2*_ = *4.0* *mm,* respectively, which appears to be largely due to differences in peripheral densities (Fig. 2e). For S-cones, however, there was no difference; in both young and old animals the S-cone density fell to half its peak value at 1.5 mm (Fig. 2f).

To estimate the aggregate density of each cone class in each animal, numerical integration over the exponential decay models was computed. This step permitted fair comparison of cone density between young and old animals, even in the presence of minor variations in the rate of exponential decay with eccentricity. The resulting values are shown in Table 1. Two one-way ANOVAs were performed: first between the old and young aggregate S-cone densities, and second between the old and young aggregate M/L-cone densities. In neither case was a significant difference in density detected (p = 0.88 and p = 0.71, respectively).

**Figure 3:**
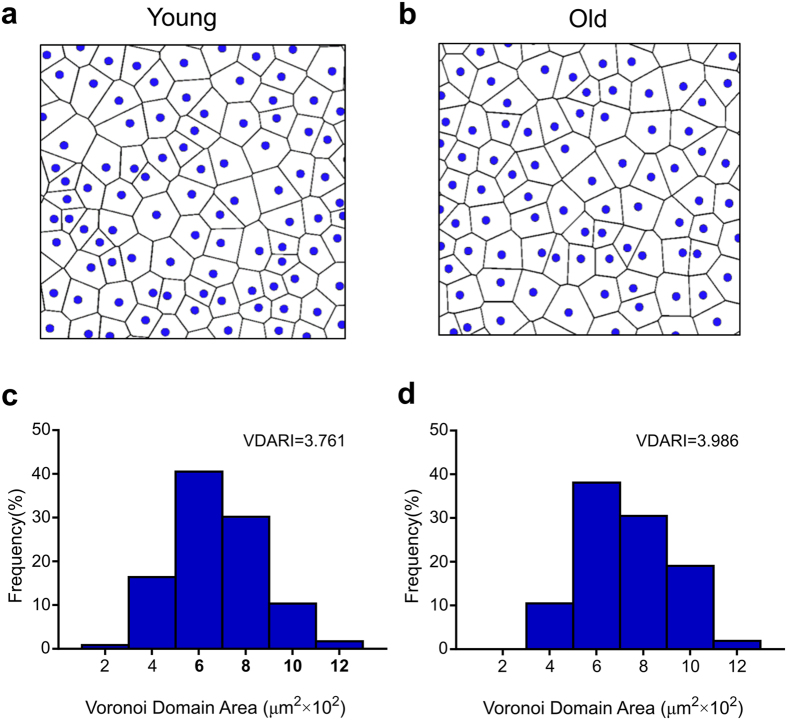
Voronoi domain analysis provides both diagrams and metrics of mosaic regularity. (**a**) and (**b**). Voronoi diagrams of S-cones in 200 × 200 μm regions of young (**a**) and old (**b**) retinae, at 1.07 mm and 1.17 mm eccentricity respectively. For each S-cone, a polygonal domain was created by identifying pixels that are closer to it than any other S-cone. The two diagrams are qualitatively similar. (**c**) and (**d**). Frequency distributions of Voronoi domain area, for the diagrams shown in A and B, respectively. The distributions are similar, echoed by the small difference between the Voronoi domain area regularity indices (VDARI), which is the area mean divided by the area standard deviation, for each sample. Together, these data show that S-cone regularity is not impacted by age, which implies no age-related loss in S-cones since such loss would result in a loss of regularity.

Because the S-cone distribution in the macaque retina is highly regular (nonrandom), loss of S-cones would result in increased disarray. In order to test the hypothesis that while S-cone function may be diminished by age, the cells are not lost in significant numbers, Voronoi domain analysis^22^ was used to quantify disarray in the S-cone mosaics. The Voronoi diagrams partitioned the image into regions, based upon proximity of the nearest S-cone. Variance among the areas or effective radii of these regions is an indication of mosaic disarray. There was no difference in these measures of disarray between the two age groups, irrespective of retinal location, confirming that no appreciable S-cone loss occurred with ageing (Fig. 3).

In spite of declining cone function^8^, these old world primates have no significant age-related reduction in cone numbers. This is particularly unexpected for functions mediated by S-cones in ageing and disease, where age-related cell loss might have been expected^9^,^23^,^24^. It could be argued that because our analysis was confined to the horizontal meridian we may have missed aged related cone loss in other areas. However, we know of no evidence for specific regional loss and saw no evidence for it when examining the retinae used in this study.

## Discussion

Human anatomical data for the ageing cone population is mixed with seemingly inconsistent evidence for both preservation and age-related loss^4^,^25^. Complicating matters further, is a recent study in mice that showed significant cone loss in the first year of life that was relatively biased towards loss of the M/L-cone population. This early cone loss preceded loss within the rod population^26^. However, there are fundamental differences in the cone populations and their relative distribution between mouse and primate that may undermine the value of a direct comparison^26^. In light of this, it may be argued that we lack evidence for a decline in S-cone mediated vision in non-human primates, but we have specifically sourced old world primates to ensure that our model is as close to the human as possible, and as such, likely to reflect the age related changes found in humans. In spite of this, it is possible that there are differences in retinal ageing between humans and primates that our data have failed to reveal. A further qualification is that we need to stress that our animal numbers in each of the two groups are relatively low and as such not amenable to statistical analysis. Lack of difference is not evidence for equality. Consequently, our results should be regarded with caution.

In primates no previous attempt has been made to count the separate S- and M/L-cone populations over age. As S-cones form only about 10% of the total cone population in these animals^27^, significant changes could occur in their number that would easily be missed in counts of the overall cone population. Further, in the case of human retinae, tissues inevitably come from a heterogeneous population in which there has been differential exposure to environmental factors that may modulate cell survival, such as poor diet and smoking. In contrast, our primate data are derived from animals that were not exposed to such factors. Poor diet and smoking are risk factors in age-related macular degeneration (AMD), which is the largest cause of visual loss in humans over 60 years of age in the western world. But studies of photoreceptor loss in AMD may be relevant to our findings, as with the spread of degeneration cones appear to remain while rods are lost, consistent with the notion that these cells are relatively robust in both ageing and disease^28^,^29^.

S-cone function declines with age in humans and while age-related brunescence of the lens selectively attenuates short-wavelength light, it does not explain all of the age-related changes in S-cone sensitivity^30^. There is a slowing of the S-cone pathway with age even after compensating for changes in the lens^24^. In both humans and old world primates, colour vision is substantially the same^16^,^17^. S-cone function is known to be selectively lost in specific metabolic conditions, which may highlight potential mechanisms of dysfunction. In each case where S-cone function declines there is a significant association with restricted metabolism, which is important in the retina as photoreceptors have the greatest energy demands in the body^31^. Hence, reduced S-cone function is found in diabetes^9^, where access to glucose is compromised. Likewise, when oxygen is reduced S-cone function declines^32^. Oxygen is critical for ATP production in mitochondria, which are densely packed in photoreceptor inner segments^33^. Other pathologies where there is evidence of selective functional decline of S-cones include retinal detachment^34^ and macular degeneration^35^,^36^,^37^, and these also have a direct link with oxygen. In retinal detachment, the outer retina is separated from its choroidal blood supply and becomes hypoxic. In macular degeneration there is deposition of extracellular material under the retinal pigment epithelium (RPE) that restricts the passage of oxygen and metabolites to photoreceptors.

These pathologies may cast light on age-related changes that selectively impact on S-cones. With normal ageing, Bruch’s membrane, which sits between the choroidal blood supply and the RPE thickens due to the accumulation of extra-cellular deposits, and consequently the outer retina becomes increasingly hypoxic^38^,^39^. If S-cones lack adaptive flexibility to respond to this they will suffer functional decline, although this appears to not result in cell death. The fact that in ageing such cells do not die implies that there may be an opportunity of restoring aged functional decline in this functionally vulnerable but anatomically robust cell type.

## Methods

### Retinal tissue

Ocular tissues were acquired from *Macaca fasciularis* from an established colony under U.K. Home Office regulation. Eyes were retrieved at death following sedation with ketamine and overdose of intravenous sodium pentobarbital. The primary purpose of animal usage was different from the aims of this study and eyes were only retrieved after death. Eyes (N = 6, 3 young at 6 years and 3 old at 17 years) were removed and placed in 4% paraformaldehyde for approximately 24 h. They were then washed in phosphate buffer. The anterior eye was removed and the retina dissected free as a whole mount. The temporal retina running from the optic nerve head to the periphery in a strip approximately 1 cm wide was dissected. This was permeabilised in 3% Triton X-100 in PBS containing 5% normal donkey serum (NDS) (Jackson Labs, USA) followed by primary antibody incubation with S-opsin (1/1,000) (goat polyclonal, SC-14363, Santa Cruz Biotecnhology, Inc., USA) and M/L-opsin (1/600) (rabbit polyclonal, AB5405, EMD Millipore, Temecula, California, USA) in PBS 1% NDS at room temperature (RT). Retinal strips were washed in phosphate buffered saline before incubation with secondary fluorescent labelled antibodies (1/2,000) for 2 h (donkey anti-rabbit Alexa Fluor 568 (A10042) and donkey anti-goat Alexa Fluor 488 (A11055), Invitrogen, Belgium). Strips were washed with PBS and Tris-buffered saline (TBS) and then mounted in Vectashield (Antifade Mounting Media, H1000, Vectashield, Burlingame, California, USA), coverslipped and sealed.

The temporal retina segments were viewed at X20 under epifluorescence. Images were captured at 1,280 × 1,024 pixels using a Digital Eclipse DXM 1200 camera (Nikon, Japan). These formed a continuous corridor from the fovea towards the temporal periphery over about 15 mm. Images were stitched together. The tissue outlines were traced on a systematic random grid placed over the photographed elements. Counts of labeled cones were undertaken in a frame of 200 × 200 μm in the upper right corner of each square. 597 sites were included in the sampling area across the retinae with an average of 99 ± 6.4 (SEM) counting sites per retina. Sites were counted only where cones were arranged in a matrix-like pattern and the image was clear and undamaged. The density of S-cones and M/L cones cells was determined at each sampled site, as the number of cells divided by the area counted at that site.

All general chemicals were acquired from Sigma Aldrich (Dorset, United Kingdom).

### Spatial analysis

S-cones were automatically identified in the anti-S-opsin stained images (the green channel in the RGB images shown in Fig. 1) as follows. First, the fundamental spatial frequency of the S-cones was determined from the spatial power spectrum (2D FFT). Next, using this frequency a custom bandpass filter was used to smooth the image, followed by thresholding the image above the noise floor. The resulting image contained easily identifiable blobs corresponding to the S-cones, the centers of mass of which were taken to be the S-cone coordinates.

S-cone coordinates were used to generate Voronoi diagrams using the Qhull^40^ and Fortune’s^41^ algorithms, implemented in Python, where each resulting Voronoi domain consists of those points in the image closer to the domain’s S-cone anchor than any other S-cone. Each domain consists of a polygon, and domain areas were computed using the shoelace formula (or surveyor’s formula). Variance among the domain areas was computed for all images (Voronoi domain area regularity index, or VDARI) which is an indication of the uniformity of the S-cones’ spatial distribution.

## Additional Information

**How to cite this article:** Weinrich, T. W. *et al*. No evidence for loss of short-wavelength sensitive cone photoreceptors in normal ageing of the primate retina. *Sci. Rep.* **7**, 46346; doi: 10.1038/srep46346 (2017).

**Publisher's note:** Springer Nature remains neutral with regard to jurisdictional claims in published maps and institutional affiliations.

## Figures and Tables

**Table 1:** Mean cone densities grouped by age and cone spectral class.

	S-densities (cones/mm^2^)		M/L-densities (cones/mm^2^)	
	Young	Old	Young	Old
	371	478	4553	4,217
	431	392	4162	4,160
	378	283	4387	4,530
**Mean**	**393**	**384**	**4368**	**4,302**

Cone densities derived from exponential models by interpolating cone density over the central 20 mm.
